# Thioredoxin Interacting Protein (TXNIP) Is Differentially Expressed in Human Tumor Samples but Is Absent in Human Tumor Cell Line Xenografts: Implications for Its Use as an Immunosurveillance Marker

**DOI:** 10.3390/cancers12103028

**Published:** 2020-10-18

**Authors:** Joana Schröder, Udo Schumacher, Lukas Clemens Böckelmann

**Affiliations:** 1 Institute of Anatomy and Experimental Morphology, Center for Experimental Medicine, University Cancer Center Hamburg, University Medical Center Hamburg-Eppendorf, 20246 Hamburg, Germany; joana_schroeder@web.de (J.S.); uschumac@uke.de (U.S.); 2 HanseMerkur Center for Traditional Chinese Medicine at the University Medical Center Hamburg-Eppendorf, 20251 Hamburg, Germany; 3 Department of Oncology, Hematology and Bone Marrow Transplantation with Section Pneumology, University Cancer Center Hamburg, University Medical Center Hamburg-Eppendorf, 20246 Hamburg, Germany

**Keywords:** cancer, immunotherapy, reactive oxygen species, thioredoxin, thioredoxin interacting protein, tissue array, tumorigenesis, xenograft

## Abstract

**Simple Summary:**

The metabolic protein TXNIP plays a crucial role in various cellular processes. Abnormal TXNIP levels are notable, e.g., in type II diabetes, cardiovascular diseases, and tumors. Using immunohistochemical staining for TXNIP in different tumor entities, we give new insights of TXNIP expression on the protein level. In human tumors, staining intensity inversely correlated with aggressiveness of the tumor entity. In contrast, human tumor cell lines grown in mice (xenografts), consistently revealed no staining. Hence, loss of TXNIP suggests a critical role for the development of tumors in xenografts. Furthermore, we investigated TXNIP staining of immunocompetent cells in the proximity of the xenograft tumor tissue. Our findings demonstrate that TXNIP downregulation is a common feature in human tumor xenograft models. Subsequently, TXNIP expression might be used to monitor the functional state of tumor-infiltrating leukocytes in tissue sections and may help to predict response to modern immune therapy.

**Abstract:**

Thioredoxin interacting protein (TXNIP) is a metabolic protein critically involved in redox homeostasis and has been proposed as a tumor suppressor gene in a variety of malignancies. Accordingly, TXNIP is downregulated in breast, bladder, and gastric cancer and in tumor transplant models TXNIP overexpression inhibits growth and metastasis. As TXNIP protein expression has only been investigated in few malignancies, we employed immunohistochemical detection in a large multi-tumor tissue microarray consisting of 2,824 samples from 94 different tumor entities. In general, TXNIP protein was present only in a small proportion of primary tumor samples and in these cases was differently expressed depending on tumor stage and subtype (e.g., renal cell carcinoma, thyroid cancer, breast cancer, and ductal pancreatic cancer). Further, TXNIP protein expression was determined in primary mouse xenograft tumors derived from human cancer cell lines and was immunohistochemically absent in all xenograft tumors investigated. Intriguingly, TXNIP expression became gradually lower in the proximity of the primary tumor tissue and was absent in leukocytes directly adjacent to tumor tissue. In conclusion, these findings suggest that TXNIP downregulation is as a common feature in human tumor xenograft models and that intra-tumoral leukocytes down-regulate TXNIP. Hence TXNIP expression might be used to monitor the functional state of tumor-infiltrating leukocytes in tissue sections.

## 1. Introduction

Oxidative stress, which is the excessive accumulation of reactive oxygen species (ROS), is an important factor in cancer development and progression [1]. Therefore, cells provide specific antioxidant defense systems to scavenge ROS and to limit damage to proteins, nucleic acids, and lipids [1]. One of such systems is the thioredoxin system, which includes thioredoxin (TRX), thioredoxin reductase (TrxR), α-nicotinamide adenine dinucleotide phosphate (NADPH), and thioredoxin interacting protein (TXNIP) [2,3]. To alleviate oxidative stress, TRX transfers reducing equivalents to oxidized proteins in the cell. Oxidized TRX, in turn, is regenerated by TrxR via the expenditure of NADPH (summarized in Figure S3) [2].

TXNIP was initially described as 1,25-dihydroxyvitamin D3-upregulated protein 1 (VDUP1) in human promyelocytic leukemia HL-60 cells [4]. It is ubiquitously expressed and highly conserved across different species [5]. TXNIP belongs to the α-arrestin protein family and is so far the only family member known, to bind TRX [6,7]. TXNIP acts as a negative modulator of TRX by binding to the catalytic center in its reduced state forming a disulfide [8].

Excessive ROS as well as many apoptotic stimuli induce rapid upregulation of TXNIP, which will ultimately lead to cell apoptosis [9,10]. TRX itself is an inhibitor of apoptosis stimulating kinase 1 (ASK1), which triggers the execution of programmed cell death by activation of c-Jun N-terminal kinase (JNK) and p38 mitogen-activated protein kinase (MAPK) pathways [11]. TXNIP competes with ASK1 to bind to TRX and therefore facilitates an increase in the availability of activated pro-apoptotic ASK1 [12]. Further, TXNIP positively regulates p53 activity in hematopoietic cells [13]. Thus, it was speculated that TXNIP functions as a tumor suppressor gene (supporting experimental evidence summarized in Table S1). Indeed, TXNIP was found to be downregulated in hepatocellular carcinoma (HCC) in a majority of patients [14]. TXNIP-KO mice are more susceptible to HCC and TXNIP-deficient tumors proliferate significantly more than their wild type counterpart [14]. Moreover, in HCC cell lines upregulation of TXNIP is implicated in the inhibition of cell proliferation and G1 cell-cycle arrest [15]. Likewise, in bladder cancer TXNIP mRNA was significantly decreased in 39 human bladder cancers as compared to 6 normal samples and could be inversely correlated to the grade and stage of bladder cancer [16].

Subsequent studies fostered the role of TXNIP in cancer pathophysiology and therapy. The histone deacetylase inhibitor suberoylanilide hydroxamic acid increases TXNIP expression, thereby causing ROS accumulation and apoptosis induction in solid tumors and leukemias, but not in normal cells [17].

Interestingly, TXNIP was also implicated in TRX-independent functions, especially in glucose and lipid metabolism. Insulin production in β-cells, glycolysis, and cellular glucose uptake haven been linked to TXNIP function as well as cellular immunity [2]. TXNIP-deficient mice, for example, lack functional natural killer (NK) cells [18] and TXNIP seems to be a prerequisite in the transformation of naïve T-cells into their activated state by increased glucose uptake and metabolism [19]. Furthermore, TXNIP stimulates cellular inflammatory responses by activating nod-like receptor protein 3 (NLRP3) inflammasome and maturation of interleukin-1β [20,21]. NLRP3 inflammasome activation is additionally augmented by TXNIP-mediated nuclear factor kappa B (NF-κB) signaling, which is a key mediator in inflammatory response [22].

Given the bifunctional nature of TXNIP and the diverse functions it can execute in tumorigenesis (see also Figure S4), its expression might differ dependent on tumor origin and tumor subtype. Therefore, we detected TXNIP protein expression immunohistochemically in a large multi-tumor tissue microarray (TMA). Furthermore, we analyzed TXNIP expression in widely used tumor cell lines taken directly from 2D-culture and in archival mouse xenograft tumor tissues.

## 2. Results

### 2.1. TXNIP Expression in Primary Human Tumor Samples

**Figure 1:**
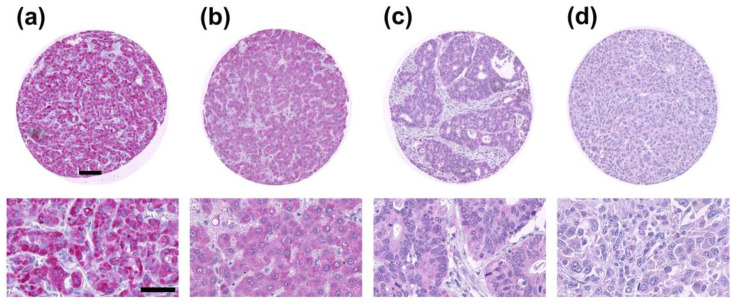
Representative images of thioredoxin interacting protein (TXNIP) immunostaining results on multi-tumor tissue micro array (TMA). (**a**) Strong TXNIP protein expression in an oncocytoma sample, (**b**) moderate TXNIP protein expression in a hepatocellular carcinoma sample, (**c**) weak TXNIP protein expression in a mucinous ovarian cancer sample, (**d**) negative TXNIP protein expression in an anaplastic thyroid cancer sample. Staining with Permanent Red. Scale bar (upper panel) = 100 µm, scale bar (lower panel) = 50 µm.

2,824 of 3,662 tumor samples were interpretable in our TMA analysis. Non-informative cases (*n* = 838; 23%) were due to lack of tissue samples or absence of unequivocal cancer tissue in the respective TMA spot. In the total cohort, negative TXNIP expression was found in 73%, weak TXNIP expression was found in 14% of all cancers, moderate TXNIP expression was found in 8%, and strong TXNIP expression was found in 5% of all cancers (Table 1). Representative IHC images of negative, weak, moderate, and strong TXNIP protein expression are shown in Figure 1.

### 2.2. TXNIP is Differently Expressed in Tumor Subtypes

**Figure 2:**
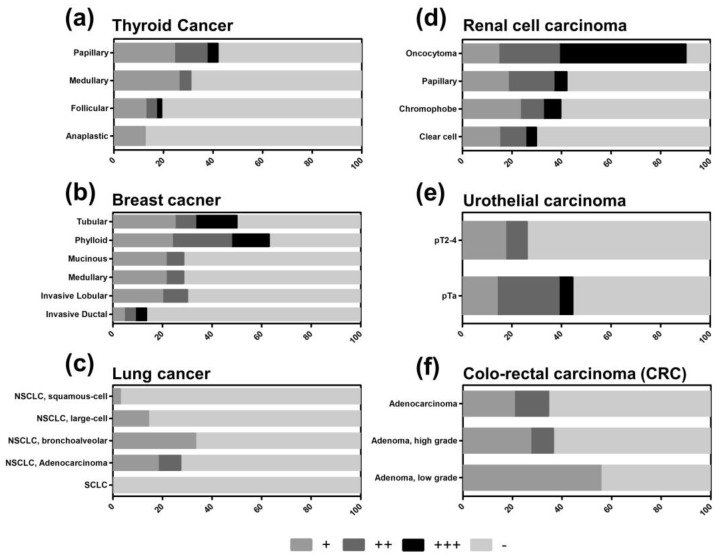
Differential expression of thioredoxin interacting protein (TXNIP) in selected tumor types and subtypes on multi-tumor TMA. (**a**) Thyroid cancer subtypes, (**b**) breast cancer subtypes, (**c**) lung cancer including non-small cell lung cancer (NSCLC) subtypes and small-cell lung cancer (SCLC), (**d**) renal cell cancer, (**e**) urothelial cancer (pTa = non-invasive papillary carcinoma, pT2-4 = invasive carcinoma), and (**f**) colo-rectal cancer (CRC). ‘−‘ = negative, ‘+’ = weak, ‘++’ = moderate, and ‘+++’ = strong TXNIP expression.

TXNIP was proposed to be a tumor suppressor [23], which can be recapitulated for some tumor entities analyzed on our TMA. In thyroid cancer, for example, the total proportion of TXNIP-positive tumors is higher in papillary carcinomas (41.9%), which are associated with the most favorable prognosis amongst thyroid cancer subtypes (Figure 2a). Follicular carcinomas of the thyroid gland have a less favorable prognosis and display a considerably lower percentage of TXNIP-positive tumors (19.2%). In contrast, TXNIP was only weakly expressed in 12.5% of undifferentiated anaplastic carcinomas having a median survival of only months [24].

Similar results were obtained for breast cancer, renal cell carcinoma, and urothelial carcinoma. In breast cancer, prognostically favorable tubular carcinomas and the heterogeneous subgroup of phyllodes tumors—with a wide range from benign over borderline to malignant types—showed a distinct higher percentage of total TXNIP-positive tumors (50.0% and 63.0%, respectively) compared to the prognostically more unfavorable other subtypes (Figure 2b). Interestingly, in invasive ductal carcinomas, only 13.6% of tumors displayed TXNIP protein expression but retained a diverse expression pattern ranging from weak to high. In kidney cancer, benign oncocytomas are TXNIP-positive in most cases (90.2%), while TXNIP expression got lost in malignant renal cell carcinomas (ranging from 29.8 to 42.1%, Figure 2d). Interestingly, in urothelial carcinomas, the proportion of TXNIP-positive tumors correlates with tumor stage. In urothelial pTa tumors, 44.4% of tumors displayed positive TXNIP expression, while among pT2-4 tumors only 26.1% were positive with complete loss of high TXNIP expression (Figure 2e).

In contrast, colo-rectal cancer specimens showed no clear patterning of TXNIP expression: TXNIP-positive tumors were detected in 34.5% of adenocarcinomas, 36.4% of high-grade adenomas, and some weak expression of 55.6% in low-grade adenomas (Figure 2f).

**Figure 3:**
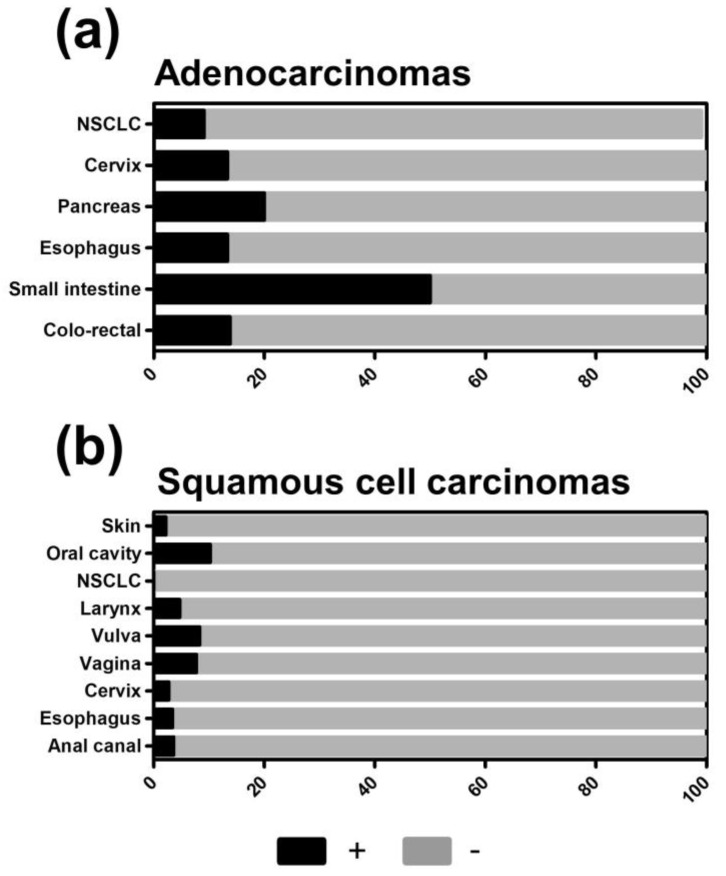
Percentage of thioredoxin interacting protein (TXNIP)-positive tumor samples of all tumor samples in adenocarcinomas (**a**) and squamous cell carcinomas (**b**) on multi-tumor TMA. ‘+’ = high, ‘−‘ = low TXNIP protein expression. NSCLC = Non-small cell lung cancer.

For further analysis, we dichotomized our results into a TXNIP low group (negative and weak expression) and a TXNIP high group (medium and high expression) to more clearly expose those tumors and subtypes that downregulate or lose TXNIP expression. Accordingly, low TXNIP expression was found in 12.6% of all cancers, while high TXNIP expression was found in 87.4% of all cancers. When comparing adenocarcinomas with carcinomas derived from squamous cells, adenocarcinomas demonstrated higher numbers of TXNIP-positive tumors (Figure 3a,b). In non-small cell lung cancer (NSCLC), for example, a proportion of adenocarcinomas showed higher TXNIP levels but not any squamous cell carcinomas (Figure 2c and Figure 3a,b).

### 2.3. TXNIP Expression in Mouse Xenograft Tumors

Tumor models, especially mouse xenografts, are of increasing importance in cancer research, but tumor biology might differ significantly from patient tumors. We, therefore, detected TXNIP protein expression in human cancer cell lines and paired mouse xenograft tumors. TXNIP expression was detected in HT-29 and HCT 116 cells (both colon carcinoma), DU4475 (breast cancer), and PaCa 5072 and PaCa 5061 cells (both pancreatic carcinoma). In all other cell lines tested, TXNIP expression was immunohistochemically absent (Table 2). Interestingly, TXNIP expression was absent in all mouse xenograft tumors tested (Table 2).

### 2.4. TXNIP Expression in Tumor Infiltrating Leukocytes

**Figure 4:**
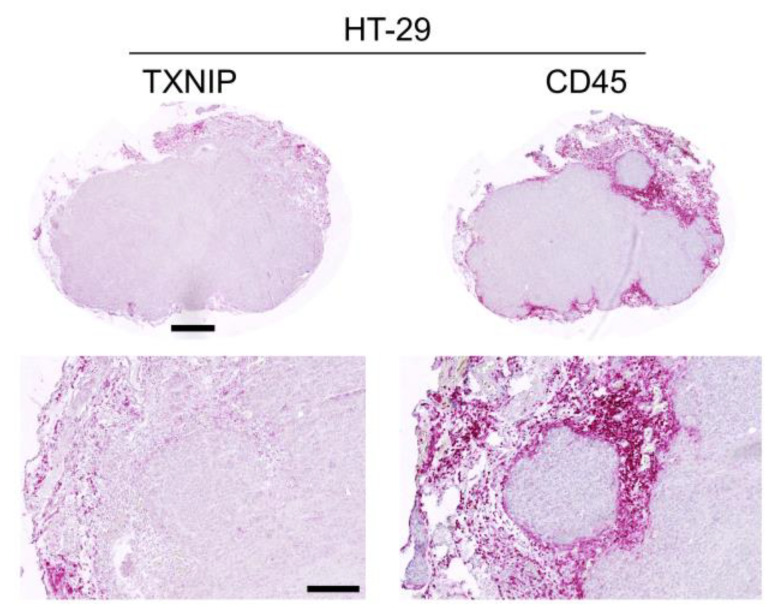
Thioredoxin interacting protein (TXNIP) and CD45 immunostaining results in a HT-29 mouse xenograft tumor. Scale bar (upper panel) = 500 µm, scale bar (lower panel) = 200 µm.

**Figure 5:**
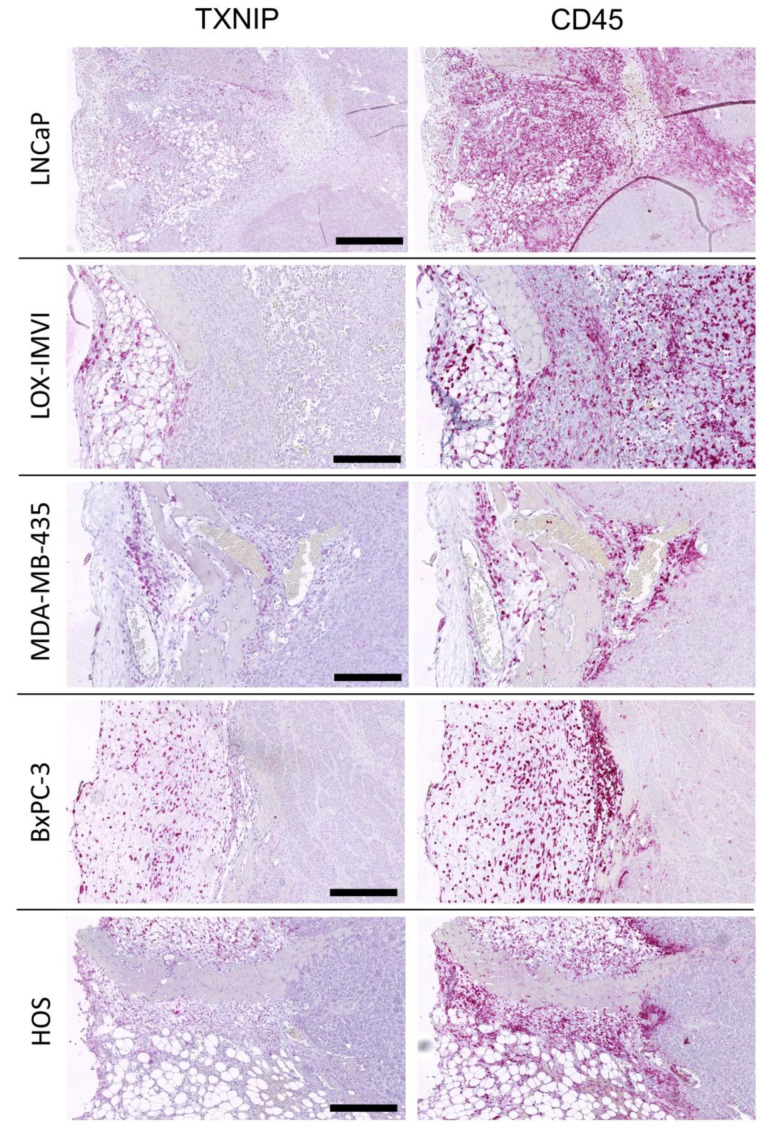
Thioredoxin interacting protein (TXNIP) and CD45 immunostaining results in mouse xenograft tumors derived from different tumor cell lines. Scale bar: LNCaP = 1000 µm, LOX-IMVI = 200 µm, MDA-MB-435 = 200 µm, BxPC-3 = 500 µm, HOS = 200 µm.

**Figure 6:**
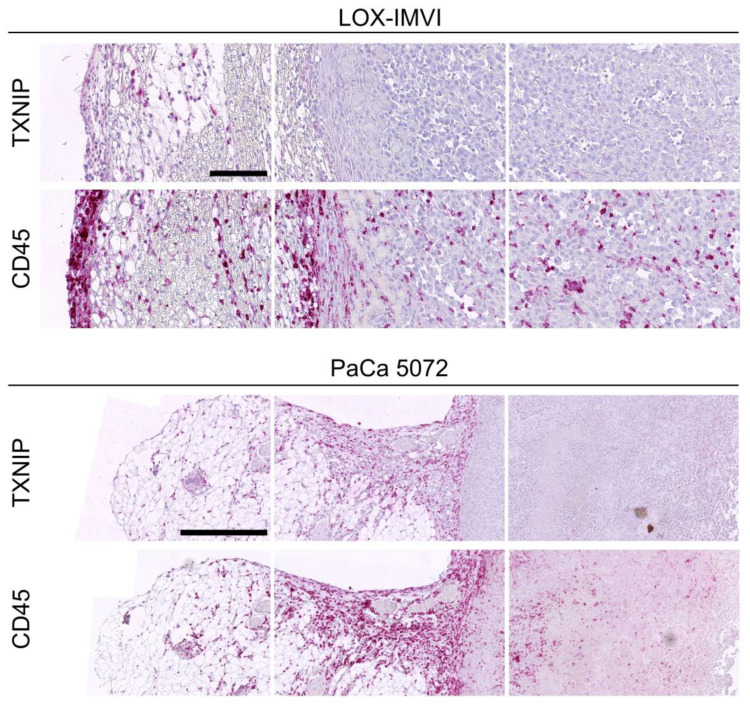
Downregulation of thioredoxin interacting protein (TXNIP) in intra- and peri-tumoral leukocytes. Scale bar: LOX-IMVI = 100 µm, PaCa 5072 = 500 µm.

We further evaluated TXNIP expression in peri- and intratumoral leukocytes as TXNIP might play a role in immune cell activation [19]. We immunohistochemically detected leukocytes by their CD45 surface expression. As the antibody used to detect human TXNIP cross-reacted with murine TXNIP, infiltrating murine CD45+ leukocytes were TXNIP positive as well, thereby serving as an internal positive control. Among the different xenograft tumors, the numbers of tumor infiltrating CD45+ leukocytes differed between 4.5% in MeWo melanoma xenograft tumors and 29.9% in BxPC-3 pancreatic cancer tumors with pancreatic cancer also being the entity with the highest leukocyte infiltration (Table 2). While the (human) tumor cells lacked any TXNIP expression, murine TXNIP expression was absent in intra- and peri-tumoral leukocytes but became gradually positive in leukocytes residing in the outer margins of the surrounding connective tissues (Figure 4, Figure 5 and Figure 6).

Leukocyte infiltration was also assessed in the patient-derived tumor samples of the TMA. The percentage of tumor samples with TXNIP positive versus negative leukocytes in the connective tissues was assessed (Table 1). The results vary between 80% in prostate adenocarcinoma samples and 0% in tumors of the adrenal gland. The informative value, however, might be limited, because, due to the production process of the TMA, the proportion of connective tissues per spot was only small.

## 3. Discussion

TXNIP serves in different cellular functions, especially in glucose metabolism and redox homeostasis [9,11,25,26], and had initially been implicated to play an important pathophysiological role in various diseases including diabetes, inflammation, and arteriosclerosis [21,27]. However, in recent years, TXNIP gained attention in tumor biology as well as the redox state of a cell critically affects induction of apoptotic pathways [9,11]. TXNIP was suggested to act as a tumor suppressor protein and was found to be downregulated in several cancers [16,28,29,30]. So far, almost all investigations on the role of TXNIP in cancer, whether in clinical human tumor tissues or in human cancer cell lines, were focused on quantification of mRNA and not on TXNIP protein expression (reviewed by Zhou et al.) [31]. The lack of actual protein expression data is critical for the interpretation of the functional role of TXNIP in tissues as Yan et al. found TXNIP protein to be downregulated by miRNA373 in the human breast cancer cell line MCF7. Importantly, this effect was mediated through translational inhibition while mRNA levels of TXNIP were not affected [32]. Similar results have been obtained by Elgort et al., demonstrating that TXNIP downregulation is important in cell cycle progression and that TXNIP protein level is reduced prior to mRNA level during the early G1 phase [33]. These findings imply that mRNA quantification may underrate the importance of TXNIP downregulation in cancer progression and gives a rationale for studying TXNIP expression at the protein level.

Several studies concluded, based on experiments between TXNIP proficient and deficient cell line pairs, that TXNIP is a tumor suppressor (supporting evidence summarized in Table S1). As TXNIP protein is downregulated or lost in a high proportion of tumors across all entities and subtypes, our results are at least supportive of this possibility. Our findings are further corroborated by in silico analysis of The Cancer Genome Atlas (TCGA) gene expression profiles across tumor samples and paired normal tissues from 31 tumor entities. In 16 out of 28 tumors TXNIP expression levels were significantly reduced compared to normal tissue and in the remaining 12 tumors a consistent tendency was evident (Figures S1 and S2). In our study, however, the remaining TXNIP-positive tumors showed marked heterogeneity in TXNIP expression levels ranging from low over medium to high. This pattern, for example, was found in renal cell carcinoma, thyroid cancer, breast cancer, and ductal pancreatic cancer. Therefore, further investigations on TXNIP expression on the tissue protein level may be useful to determine whether TXNIP can constitute different subsets of patients and might be a prognostic marker and to further delineate its role as a possible tumor suppressor.

Further, TXNIP is an endogenous inhibitor of TRX activity and expression. The TRX/TXNIP system (sometimes referred to as ‘redoxisome’) is an indispensable modulator of cellular redox state and ROS signaling. TRX and TXNIP exhibit reciprocal expression patterns, which not only holds true in the context of ROS production but also for several other biological events, such as apoptosis, inflammation, or neurodegeneration (reviewed by Yoshihara et al. [34]). Therefore, it is interesting to note that, in contrast to TXNIP, TRX is upregulated in various cancers [35]. An increase in TRX and decrease in TXNIP expression might be the two sides of the same medal, which was demonstrated in cell lines [8], as well as in clinical samples of gastric and colorectal cancer patients [29,36].

Pharmacological regulation of the TRX/TXNIP system might be an effective therapeutic approach. There is clear evidence that inhibition of TRX and/or TrxR exhibit anti-tumor activity in vitro and in vivo [3]. Inhibitors, such as auranofin or PX-12, exhibit anticancer activity in animal models and are approved for clinical cancer trials (NCT01737502, NCT03456700, NCT00736372, NCT00417287). For TXNIP, on the other side, expression inducing substances such as D-Allose or the HDAC inhibitor suberoylanilide hydroxamic acid hydroxamic are promising candidates [3].

TXNIP has emerged as a key negative regulator of cellular glucose uptake [7,25,26,37] and many tumors rely on glucose fermentation instead of mitochondrial oxidative phosphorylation (also known as aerobic glycolysis or the ‘Warburg effect’). This switch in cancer cell metabolism fuels new biosynthesis by increasing the availability of metabolites from glycolysis, which in turn promotes malignant growth (reviewed by Heiden et al. [38]). Thus, downregulation of TXNIP would confer a transformative advantage in cancer progression. The Warburg effect especially holds true for cancer cells grown in vitro, which might explain absent TXNIP expression in almost all tumor cell lines and all xenograft tumors. Intriguingly, glucose uptake is also a limiting factor in T Cell activation. Levring et al. found that naïve T cells express high levels of TXNIP and that tumor necrosis factor (TNF) induces a rapid downregulation of TXNIP with increased glucose uptake in T cells [19]. Immune modulatory functions of TXNIP might control immune recognition and elimination of tumor cells. In our study, TXNIP was specifically absent in intra-tumoral and directly peritumoral leukocytes, while more distant leukocytes remained TXNIP-positive. As the xenograft tumors were grown in immunodeficient mice, which lack functional T- and B- lymphocytes, the only remaining lymphocytes in these mice are NK-cells. Brodbeck et al. demonstrated, that additional knock-out of NK-cell function in mice impedes primary tumor growth much less than the growth of distant metastases [39]. This finding could be in part explained by the fact that NK-cell activity in primary human xenograft tumors could be so much down-regulated by TXNIP, that they are functionally inactive. Thus, TXNIP might display immune activation in a tumor site and may aid in predicting response to modern immune therapy.

## 4. Materials and Methods

### 4.1. Cell Lines and Cell Culture

The human small cell lung cancer cell lines H69AR1, H69AR3, NCI-H69 and SW2, human colon carcinoma cell lines HT-29 and HCT 116, human breast cancer cell lines DU4475, MCF-7 and MDA-MB-231, human melanoma cell lines FEMX-1, LOX-IMVI, MeWo, and MV3, human neuroblastoma cell lines LA-N-1 and LS, human Osteosarcoma cell lines HOS/Kelly and HOS/SK-N-SH, human pancreas cell lines PaCa 5072, PaCa 5061, BxPC-3, and PANC-1, human prostate cancer cell lines LNCap and PC-3 were analyzed. All cell lines were grown in Roswell Park Memorial Institute (RPMI-1640) medium (GibcoTM, Waltham, MA, US). For all cell lines, media were supplemented with 10% fetal calf serum (FCS) and 2mM L-Glutamine, 100 units/mL penicillin and 100 µg/mL streptomycin (GibcoTM, Waltham, MA, US). The cells grow adherently except H60AR-1 and DU4475, which were cultured in suspension. All cells were maintained in a humidified atmosphere of 95% air plus 5% CO2 at 37 °C.

### 4.2. Mouse Xenograft Tumors of Human Cancer Cell Lines

The xenografted primary human tumors grown in immunodeficient scid mice were retrieved from the files of the Institute of Anatomy and Experimental Morphology from previous experiments. Small cell lung cancer: H69AR, NCI-H69, NCI-H82, and SW2 [40]; colon cancer: HT-29 and SW2 [41,42]; breast cancer: DU4475, MCF-7, MDA-MB-231, and T-47D [41,43,44]; melanoma: FEMX-1, LOX-IMVI, MDA-MB-435, MeWo, and MV3 [43,45]; neuroblastoma: LA-N-1 and LS [46]; osteosarcoma: HOS and U2OS; pancreatic cancer: PaCa5072, PaCa5061, BxPC-3, and PANC-1 [47]; prostate cancer: DU145, LNCaP, LuCaP 23.1, PC-3, and VCaP [48].

### 4.3. Multi-Tumor Tissue Microarray (TMA)

For the production of TMAs, tissue cylinders with a diameter of 0.6 mm were punched from representative tumor or normal areas of each tissue block and brought into a recipient paraffin block. All tumor samples were obtained from the archives of the Institute of Pathology of the University Medical Center Hamburg Eppendorf. The use of archived diagnostic left-over tissues for the manufacturing of TMAs and their analysis for research purposes has been approved by local laws (HmbKHG, §12,1) and by the local ethics committee (Ethics commission Hamburg, WF-049/09). All work has been carried out in compliance with the Helsinki Declaration. Freshly cut TMA sections were immunostained on one day and in one experiment (see below).

### 4.4. Fixation, Embedding and Sectioning of Cancer Cells and Xenograft Tumors

Preparation of cancer cells as formalin-fixed paraffin-embedded (FFPE) samples was carried out as previously described [49]. Briefly, cancer cells were collected from culture flasks, fixed in formalin and then embedded into agar pellets. Agar pellets and formalin-fixed xenograft tumors were then subjected to standardized tissue infiltration using a Leica TP1020 tissue processor (Leica Biosystems, Nussloch, Germany). Subsequent paraffin embedding was performed using a Leica EG1160 Paraffin Embedding Center (Leica Biosystems, Nussloch, Germany). FFPE samples were sectioned with a thickness of 4 µm, mounted on HistoBond^®^ glass slides (Paul Marienfeld, Lauda-Königshofen, Germany) and allowed to air-dry, followed by drying in an incubator at 37 °C overnight.

### 4.5. Immunohistochemistry (IHC)

FFPE sections were de-paraffinized in two changes of xylene (5 min each) and rehydrated in a series of graded ethanol (100, 96, 70 and 50% for 5 min each). Sections were then washed in distilled water for 2 min. In case of CD45, tumor sections were heated twice in a 1:10 dilution of epitope retrieval solution (#S1699, Dako, Carpinteria, CA, USA) in a microwave oven (#YC-MG02U-S, Sharp) with 800W for 4 min each. In case of TXNIP, slides were heated in a steamer (#S2800, DakoCytomation Pascal, Carpinteria, CA, USA) at 100 °C for 20 min in epitope retrieval solution (#S1699, Dako, Carpinteria, CA, USA). After cooling-down, sections were rinsed for 5 min each with Tris-buffered saline/0.1% Tween20 (TBS-T) and TBS (pH 7.6), respectively. The following incubation steps were carried out in a moist chamber. Sections were incubated either with primary rat anti-CD45 antibody (#550539, BD PharMingen, CA, USA) diluted 1:25 in antibody diluent (#S0809, Dako Carpinteria, CA, USA) or primary rabbit anti-TXNIP polyclonal antibody (#LS-B5829, LSBio, Seattle, WA, USA) diluted 1:200 in antibody diluent for 60 min each at room temperature (RT). For isotype control rat IgG2b antibody (#400622, BioLegend, San Diego, CA, USA; diluted 1:400) or rabbit IgG antibody (#ab37415, abcam, Berlin, Germany; diluted 1:1,000) were used. After incubation, slides were rinsed twice with TBS-T as well as with TBS for 5 min each. Subsequently, slides were incubated with secondary biotin-conjugated rabbit anti-rat antibody (#312-065-048, Jackson Immuno Research Europe Ltd., Ely, Cambridgeshire, United Kingdom) or swine anti-rabbit antibody (#E0353, Dako, Carpinteria, CA, USA) at a dilution of 1:100 or 1:200, respectively, in TBS for 30 min at RT, followed by rinsing twice with TBS-T and once with TBS for 5 min each. Next, sections were treated with Vectastain^®^ ABC-AP Kit (#AK5000, Linaris, Drossenheim, Germany) according to the manufacturer’s recommendations for 30 min at RT and again washed in TBS-T and TBS as described above. Finally, alkaline phosphatase enzyme activity was visualized by incubating the sections with Permanent Red solution (#ZUC001-125, Zytomed Systems GmbH, Berlin, Germany) for 20 min and counterstained with hematoxylin for 4 seconds, with intermediate washes under running tap water (3 min) and in aqua dest (2 min). Slides were dehydrated in a series of graded ethanol (70% for 15 sec, 96 and 100% for 5 min each) and three changes of xylene (5 min each) and finally covered with Eukitt^®^ Mounting Medium (#03989, Sigma-Aldrich, Taufkirchen, Germany) and coverslips.

### 4.6. Microscopy and Image Analysis

IHC processed sections were first evaluated using a ZEISS Axiophot 2 microscope (Carl Zeiss, Jena, Germany). Digital images were obtained with a ZEISS Axio Scan Z1 slide scanner equipped with a ZEISS EC Plan-Neofluar 20 × /0,50 Pol M27 objective (Carl Zeiss, Jena, Germany) and a Hitachi HV-F20SCL camera with 1600 × 1200 pixels (Hitachi Kokusai Electric America Ltd., New York, NY, USA). For image acquisition ZEISS ZEN 2.3 software was used (Carl Zeiss, Jena, Germany). Images were further processed with netScope Viewer software (Net-Base Software, Freiburg, Germany). Image analysis of xenograft tumor sections was carried out using Fiji, an open source image processing package from ImageJ (U. S. National Institutes of Health, Bethesda, MD, USA, https://imagej.nih.gov/ij/, 1997–2018), to determine the percentage of immunocompetent cells in the following localizations: adipose tissue, tumor capsule connective tissue, intratumoral connective tissue septae and immune cells in the tumor. The area of the specific tissue was measured followed by the measurement of the area of positive cells within the tissue. Positive cells were selected with contrast threshold adjustment as follows: Hue: 196/255, Saturation: 99/255, Brightness: 0/200. Proportions of CD45-positive cells in relation to TXNIP-positive cells were calculated and are stated in percent.

TXNIP staining was observed in the cytoplasm and the staining intensity was assessed on a four-step scale from negative, weak, moderate to strong expression. Further analysis included dichotomization of staining intensities (negative to weak = ‘low’, moderate to strong = ‘high’).

## 5. Conclusions

In conclusion, this study suggests that TXNIP downregulation is as a common feature in human tumor xenograft models and provides new insights into TXNIP expression on the protein level in various tumor entities. Further, TXNIP might display immune activation in a tumor site and may aid in predicting response to modern immune therapy.

## Figures and Tables

**Table 1:** Immunohistochemical detection of thioredoxin interacting protein (TXNIP) in a multi-tumor tissue microarray.

Organ System	Tissue	Specific Tumor Entity	*n*	Negative %	Weak %	Medium %	High %	Leukocytes TXNIP Positive %
Bone		Chondrosarcoma	14	86	7	7	0	7
		Osteosarcoma	20	80	20	0	0	15
Endocrine	Adrenal gland	Adenoma	43	47	35	14	5	0
		Carcinoma	14	57	43	0	0	0
		Pheochromocytoma	30	13	20	13	53	0
	Thyroid	Adenoma	47	81	11	6	2	4
		Anaplastic carcinoma	24	88	13	0	0	13
		Follicular carcinoma	47	81	13	4	2	2
		Medullary carcinoma	42	69	26	5	0	10
		Papillary carcinoma	45	58	24	13	4	2
		Neuroendocrine tumor	27	41	33	7	19	30
Gastrointestinal	Anal canal	Squamous cell carcinoma	28	79	18	4	0	43
	Large intestine	Adenoma, low grade	18	44	57	0	0	33
		Adenoma, high grade	22	64	27	9	0	64
		Adenocarcinoma	29	66	21	14	0	52
	Small intestine	Adenocarcinoma	6	33	17	50	0	33
	Liver	Cholangiocellular carcinoma	29	55	24	17	3	10
		Hepatocellular carcinoma	41	32	42	22	5	0
	Stomach	Carcinoma, diffuse Type	34	94	6	0	0	47
		Carcinoma, intestinal Type	31	77	19	3	0	52
	Esophagus	Adenocarcinoma	30	67	20	13	0	53
		Squamous cell carcinoma	29	93	3	3	0	69
	Pancreas	Adenocarcinoma, ductal	36	61	11	17	11	67
		Adenocarcinoma, papillary	18	78	11	11	0	67
		Neuroendocrine Tumor	29	52	21	21	7	45
		Gastrointestinal Stroma tumor	37	92	5	3	0	16
Gynecologic	Cervix	Adenocarcinoma	45	73	13	11	2	42
		Squamous cell carcinoma	37	92	5	0	3	51
	Endometrium	Carcinoma, endometroid	46	74	15	7	4	30
		Carcinoma, serous	40	78	20	3	0	0
	Breast	Carcinoma, ductal (NST)	22	87	5	5	5	14
		Carcinoma, lobular	30	70	20	10	0	43
		Carcinoma, medullary	14	71	21	7	0	50
		Carcinoma, mucinous	14	71	21	7	0	21
		Carcinoma, phyllodes	46	37	24	24	15	30
		Carcinoma, tubular	12	50	25	8	17	50
	Ovaries	Brenner tumor	6	50	50	0	0	17
		Carcinoma, endometroid	28	61	21	7	11	11
		Carcinoma, mucinous	24	54	33	13	0	13
		Carcinoma, serous	41	66	20	10	5	22
	Uterus	Carcinosarcoma	47	81	13	6	0	13
		Stroma sarcoma	12	100	0	0	0	25
	Vagina	Squamous cell carcinoma	26	92	0	8	0	38
	Vulva	Squamous cell carcinoma	36	78	14	6	3	67
Hematologic		Hodgkin-Lymphoma	41	98	2	0	0	27
		Non-Hodgkin-Lymphoma	43	100	0	0	0	40
		Thymoma	26	77	19	4	0	46
Head, Chest, and Respiratory tract	Larynx	Squamous cell carcinoma	43	81	14	5	0	65
	Lung (Non-small cell)	Adenocarcinoma	33	73	18	9	0	52
		Bronchoalveolar carcinoma	6	67	33	0	0	67
		Bronchial carcinoma, large cell	21	86	14	0	0	43
		Squamous cell carcinoma	34	97	3	0	0	50
	Lung (Small cell)	Carcinoma	12	100	0	0	0	42
		Metastasis small cell carcinoma	2	100	0	0	0	0
	Oral cavity	Squamous cell carcinoma	49	67	22	4	6	57
	Pleura	Mesothelioma	24	65	13	19	3	17
	Salivary glands	Basal cell adenoma	14	43	0	43	14	7
		Parotis, pleomorphic adenoma	47	89	9	0	2	4
		Parotis, Warthin tumor	47	9	21	30	41	19
Skin		Basalioma	43	95	2	2	0	0
		Benign Naevus	27	93	4	4	0	41
		Melanoma	44	82	7	11	0	18
		Merkel cell carcinoma	38	100	0	0	0	13
		Pilomatrixoma	26	100	0	0	0	35
		Squamous cell carcinoma	45	84	11	4	0	60
Soft tissue		Angiosarcoma	27	93	0	7	0	63
		Granular cell tumor	21	100	0	0	0	76
		Giant cell tumor of tendon sheath	38	82	18	0	0	42
		Leiomyoma	50	98	2	0	0	40
		Leiomyosarcoma	48	94	6	0	0	52
		Liposarcoma	35	94	6	0	0	46
Stromal cell	Brain		20	85	15	0	0	5
	Heart		19	84	16	0	0	0
	Kidney		16	13	0	50	38	0
	Large intestine		8	63	25	13	0	0
	Liver		13	33	8	8	50	0
	Lymph nodes		9	100	0	0	0	22
	Pancreas		15	0	0	13	87	0
	Prostate		19	58	21	21	0	21
	Skin		3	100	0	0	0	33
	Thyroid		17	94	6	0	0	6
Urogenital	Kidney	Carcinoma, clear cell	47	70	15	11	4	32
		Carcinoma, chromophobe	43	61	23	9	7	2
		Carcinoma, papillary	38	58	18	18	5	13
		Oncocytoma	41	10	15	24	51	5
	Prostate	Adenocarcinoma	45	82	18	0	0	80
		Small cell	11	100	0	0	0	27
	Germ cell tumor	Carcinoma, embryonal	49	100	0	0	0	4
		Seminoma	48	100	0	0	0	4
		Teratoma	33	70	18	6	6	55
		Yolk sac tumor	45	100	0	0	0	9
	Bladder	Carcinoma, small cell	16	88	13	0	0	31
	Urothelial	Carcinoma, pTa	36	56	14	25	6	19
		Carcinoma, T2-4	46	74	17	9	0	41

**Table 2:** Expression of thioredoxin interacting protein (TXNIP) in human cancer cell lines and mouse xenograft tumors.

Tumor	Cell Line	*n*	Mean Area of Positive Cells in %			Cells in Agar	Tumor Cells
			Surrounding Tissues				
			CD45	TXNIP	TXNIP/CD45	TXNIP	TXNIP
Bronchial Carcinoma	H69AR1	4	6.4	1.7	26.6	−	−
	H69AR3	3	8.7	5.6	64.4	−	−
	NCI-H69	5	4.6	1.7	37.0	−	−
	NCI-H82	4	12.1	5.5	45.5		−
	SW2	4	5.1	3.1	60.8	−	−
Colon Carcinoma	HT-29	5	12.5	2.1	16.8	++	−
	HCT 116					+	−
Mamma Carcinoma	DU4475	2	9.6	3.6	37.5	+	−
	MCF-7	5	7	0.7	10.0	−	−
	MDA-MB-231	5	19.6	3.8	19.4	−	−
	T-47D	2	5.8	2.6	44.8		−
Melanoma	FEMX-I	5	6.3	3.6	57.1	−	−
	LOX-IMVI	5	5.2	1.3	25.0	−	−
	MDA-MB-435	5	5.3	2.4	45.3		−
	MeWo	5	4.5	2.9	64.4	−	−
	MV3	2	4.6	1.5	32.6	−	−
Neuroblastoma	LA-N-1	7	6.5	6.5	100.0	−	−
	LS	4	8.3	7	84.3	+	−
Osteosarcoma	U2OS	3	12.4	3.5	28.2	−	−
	HOS	4	8.1	3.9	48.1	−	−
Pancreas Carcinoma	PaCa 5072	4	8.9	2.8	31.5	+	−
	PaCa 5061	4	13.9	3.5	25.2	+	−
	BxPC-3	5	29.9	6.3	21.1	−	−
	PANC-1	5	12	4	33.3	−	−
Prostate Carcinoma	DU145	4	7	1.9	27.1		−
	LNCaP	4	9.9	1.9	19.2	−	−
	LuCaP 23.1	3	9.3	2.7	29.0		−
	PC-3	3	9.8	2	20.4	−	−
	VCaP	3	13.1	1.2	9.2		−

## Supplementary Materials

The following are available online at https://www.mdpi.com/2072-6694/12/10/3028/s1, Figure S1: Thioredoxin interacting protein (TXNIP) gene expression profiles across tumor samples and paired normal tissues (matched TCGA normal and GTEx data), Figure S2: Boxplot diagrams of thioredoxin interacting protein (TXNIP) mRNA expression levels of tumor samples and paired normal tissues (matched TCGA normal and GTEx data), Figure S3: The TRX/TXNIP system, Figure S4: Thioredoxin interacting protein (TXNIP) is involved in tumorigenesis and cancer progression, Table S1: Experiments investigating the consequences of altered thioredoxin interacting protein (TXNIP) expression in cancer models.
