# Supplementary material for: Thioredoxin Interacting Protein (TXNIP) Is Differentially Expressed in Human Tumor Samples but Is Absent in Human Tumor Cell Line Xenografts: Implications for Its Use as an Immunosurveillance Marker
Source: Cancers (Basel). 2020 Oct 18;12(10):3028. doi: 10.3390/cancers12103028 (PMC7603212; doi:10.3390/cancers12103028)
Supplement: Supplementary file 1 [file cancers-12-03028-s001.pdf]

*Supplemental Material*

# Thioredoxin Interacting Protein (TXNIP) Is Differentially Expressed in Human Tumor Samples but Is Absent in Human Tumor Cell Line Xenografts: Implications for Its Use as an Immunosurveillance Marker

**Joana Schröder, Udo Schumacher and Lukas Clemens Böckelmann**

**Table S1.** Experiments investigating the consequences of altered thioredoxin interacting protein (TXNIP) expression in cancer models.

| Methods | Model | Results | Reference |
|---|---|---|---|
| ▪ TXNIP deficient mice and control strain | ▪ Recombinant congenic strain HcB-19/Dem<br>▪ Parental control strain C3H/DiSnA | ▪ TXNIP deficient mice had in increased incidence of HCC<br>▪ No tumor development in control strain<br>▪ Tumor incidence increased as mice aged<br>▪ Increased p53 and $\alpha$-fetoprotein in TXNIP-deficient mice | [1] |
| ▪ TXNIP KO mice and WT mice<br>▪ Ectopic overexpression *in vitro* | ▪ C57B/6 knockout mice<br>▪ Human bladder cancer cell lines 253J, TCCSUP | ▪ Bladder carcinogenesis was accelerated in TXNIP KO mice<br>o 100% after 8 weeks in KO mice<br>o 22% after 8 weeks in WT mice<br>o ERK activation<br>▪ Overexpression in cell lines also led to ERK activation | [2] |
| ▪ TXNIP KO mice lung fibroblast cells and WT mice lung fibroblast cells | ▪ C57B/6 knockout mice | ▪ p27 is reduced in many tumors<br>▪ p27 expression was reduced in TXNIP-/- cells (post-transcriptionally)<br>▪ overexpression of TXNIP restored the JAB1-induced p27 suppression<br>▪ TXNIP -/- lung fibroblast cells in mice grew faster than their WT counterpart | [3] |

| | | | |
|---|---|---|---|
| ▪ Overexpression in an *in vivo* orthotopic tumor model<br>▪ Ectopic overexpression *in vitro* | ▪ Athymic nude mice injected with T238 QCXIP and T238 cells<br>▪ Human anaplastic thyroid cancer (ATC) cell lines TPC1, BCPAP, MDAT41, K1<br>▪ Human differentiated thyroid cancer (DTC) cell lines HTh74, T238, C643, THJ 11T, Ocut-2, 8505C | ▪ TXNIP overexpression in orthotopic tumor model attenuates growth and reduced metastatic tumor burden<br>▪ DTC cell lines have high endogenous TXNIP levels<br>▪ ATC cell lines have low/absent TXNIP expression levels<br>o TXNIP is lost in progression from well-differentiated to undifferentiated thyroid tumors<br>▪ TXNIP overexpression in ATC cell line resulted in decreased growth | [4] |
| ▪ TXNIP KO mice and WT mice<br>▪ Ectopic overexpression *in vitro* | ▪ C57B/6 knockout mice<br>▪ Human gastric cancer cell line AGS | ▪ Overall incidence of stomach tumors was higher in TXNIP KO mice compared to WT mice<br>▪ TXNIP overexpression decreased growth of cell lines infected with H. pylori (increased apoptosis) | [5] |
| ▪ Xenografts overexpressing TXNIP versus control vectors<br>▪ Ectopic overexpression *in vitro* | ▪ Athymic nude mice injected with NCI-Esc2 cells<br>▪ Human esophageal adenocarcinoma cell lines (EACC) NCI-SB-Esc1, NCI-SB-Esc2, NCI-SB-Esc3, OE33, Flo-1 | ▪ Xenografts overexpressing TXNIP were significantly smaller than tumors derived from vector controls<br>▪ Cancer cell lines (*in vitro*)<br>o TXNIP overexpression inhibited proliferation in EACC (decreased soft agar colony formation) | [6] |
| ▪ TXNIP KO and WT mice hematopoietic cells | ▪ C57B/6 knockout mice | ▪ ROS levels were elevated in TXNIP KO hematopoietic cells<br>o increased apoptosis<br>▪ TXNIP directly interacts with p53 (induction)<br>o interaction is increased by oxidative stress | [7] |
| ▪ TXNIP knockdown *in vitro* and *in vivo* | ▪ Human breast cancer tumor samples<br>▪ MMTV-Cre;$Smad4^{F/F}$;$Trp53^{F/F}$;$Cdh1^{F/+}$ mice<br>▪ Human breast cancer cell lines MCF7, T47D, MDA-MB-231 | ▪ TXNIP is downregulated in human breast cancers and animal mammary tumors on the protein level<br>o especially in aggressive phenotypes<br>▪ Degree of TXNIP expression is gradually decreased as tumors progressed from benign to malignant stages in animal mammary tumors<br>▪ TXNIP downregulation enhanced growth of breast cancer cells and breast cancer xenografts<br>o p27 is reduced | [8] |
| ▪ TXNIP KO mice and WT mice | ▪ C57B/6 knockout mice | ▪ TXNIP KO mice are suspectable to chemically induced hepatocarcinogenesis<br>o increased proliferation rate<br>o loss of TXNIP results in increased TNF-α and NF-κB activation<br>▪ TNF-α induced downregulation of TXNIP expression | [9] |

| | | | |
|---|---|---|---|
| ▪ Fe-NTA-induced renal cell carcinoma | ▪ Male Wistar rats | ▪ TXNIP was one of the major target genes in renal carcinogenesis<br>o transcriptional silenced<br>o several mutations<br>▪ Loss of TXNIP was associated with renal tubular proliferation in vivo, irrespective of the molecular mechanisms involved (neoplastic or non-neoplastic) | [10] |
| ▪ qRT-PCR measurement of mRNA | ▪ Healthy controls, AML cell lines, and primary AML lines | ▪ Repressed TXNIP expression in AML cell lines and primary AML cells | [11] |

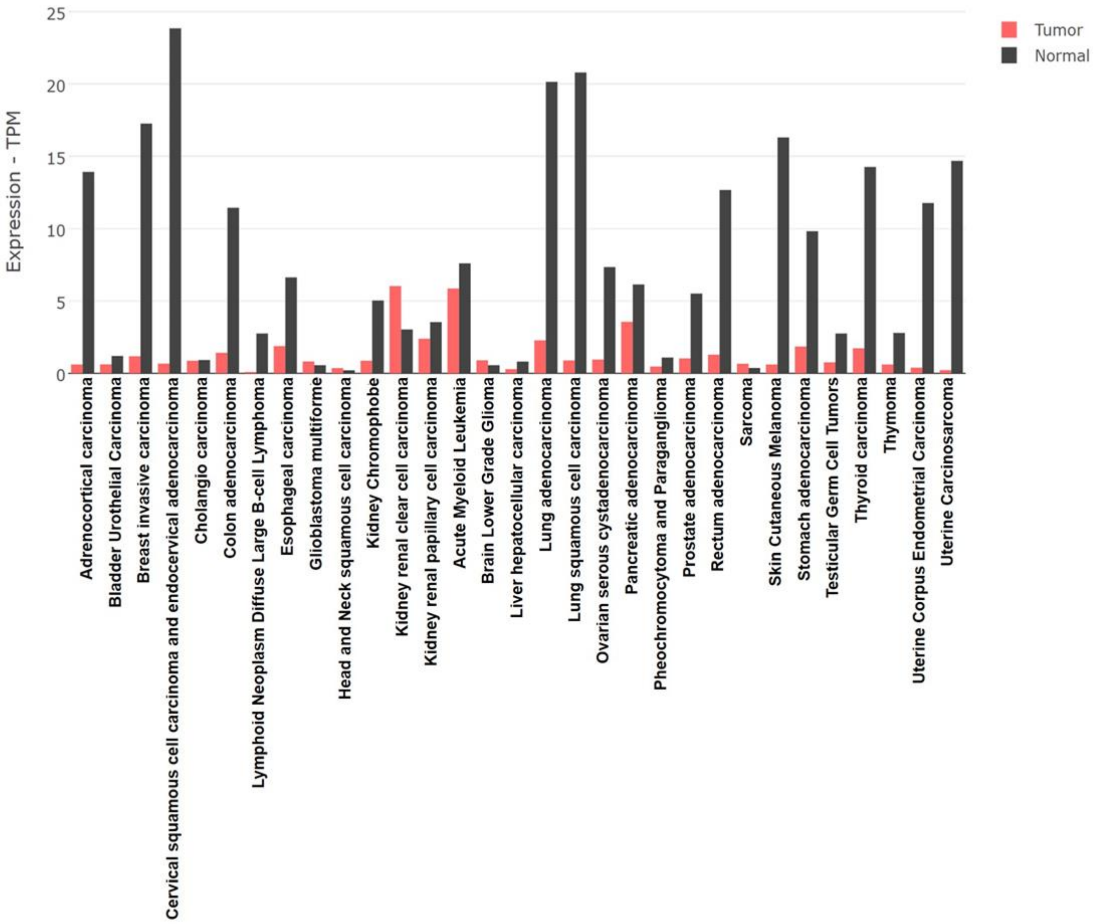


**Figure S1.** Thioredoxin interacting protein (TXNIP) gene expression profiles across tumor samples and paired normal tissues (matched TCGA normal and GTEx data). Height of bar represents the median expression of certain tumor type or paired normal tissue. TPM = Transcripts Per Million. The Cancer Genome Atlas (TCGA) data were analyzed with Gene Expression Profiling Interactive Analysis 2 (GEPIA2) online tool [12].

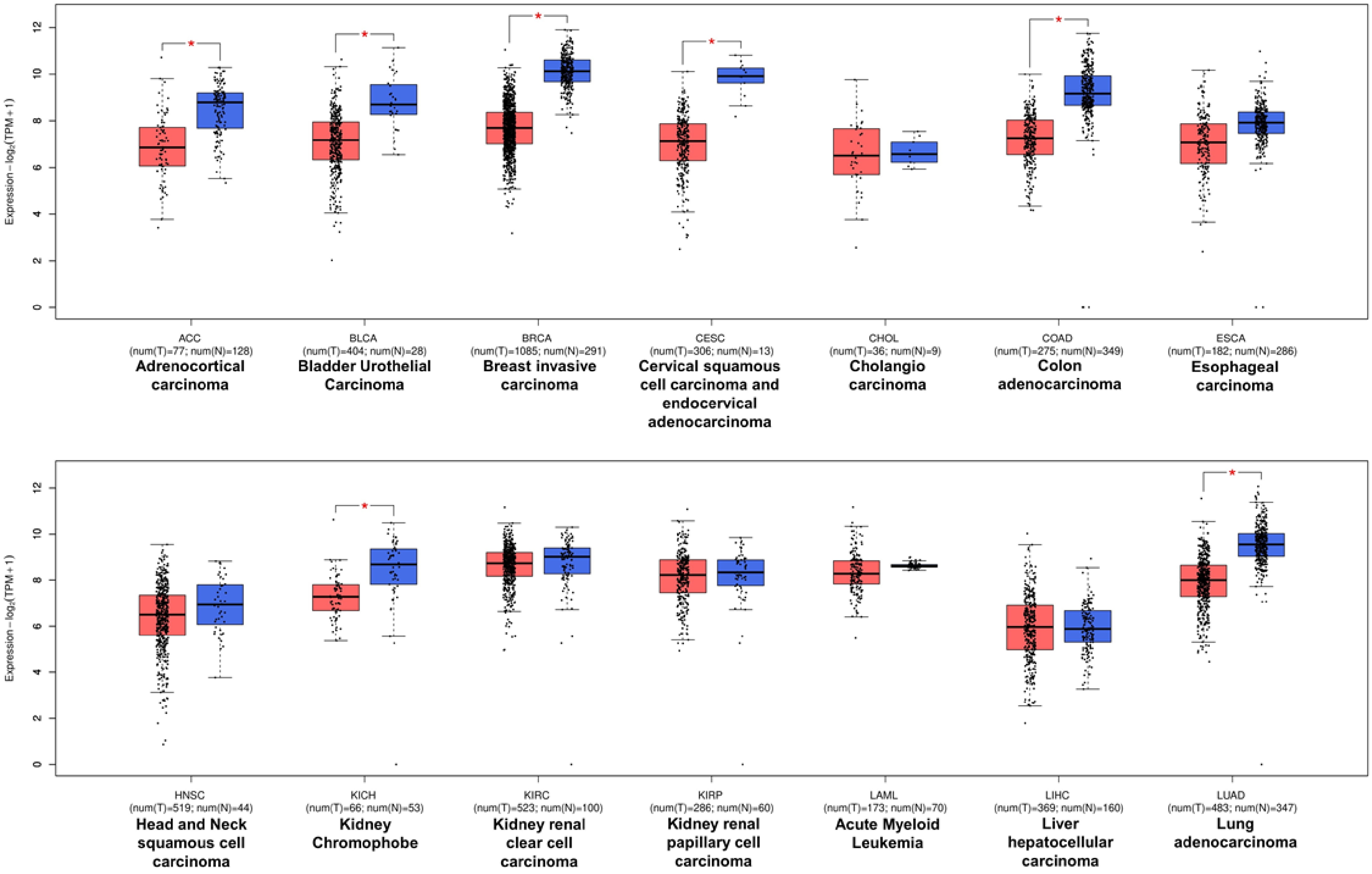
Expression – log2(TPM + 1)
ACC
(num(T)=77; num(N)=128)
Adrenocortical carcinoma
BLCA
(num(T)=404; num(N)=28)
Bladder Urothelial Carcinoma
BRCA
(num(T)=1085; num(N)=291)
Breast invasive carcinoma
CESC
(num(T)=306; num(N)=13)
Cervical squamous cell carcinoma and endocervical adenocarcinoma
CHOL
(num(T)=36; num(N)=9)
Cholangio carcinoma
COAD
(num(T)=275; num(N)=349)
Colon adenocarcinoma
ESCA
(num(T)=182; num(N)=286)
Esophageal carcinoma
HNSC
(num(T)=519; num(N)=44)
Head and Neck squamous cell carcinoma
KICH
(num(T)=66; num(N)=53)
Kidney Chromophobe
KIRC
(num(T)=523; num(N)=100)
Kidney renal clear cell carcinoma
KIRP
(num(T)=286; num(N)=60)
Kidney renal papillary cell carcinoma
LAML
(num(T)=173; num(N)=70)
Acute Myeloid Leukemia
LIHC
(num(T)=369; num(N)=160)
Liver hepatocellular carcinoma
LUAD
(num(T)=483; num(N)=347)
Lung adenocarcinoma

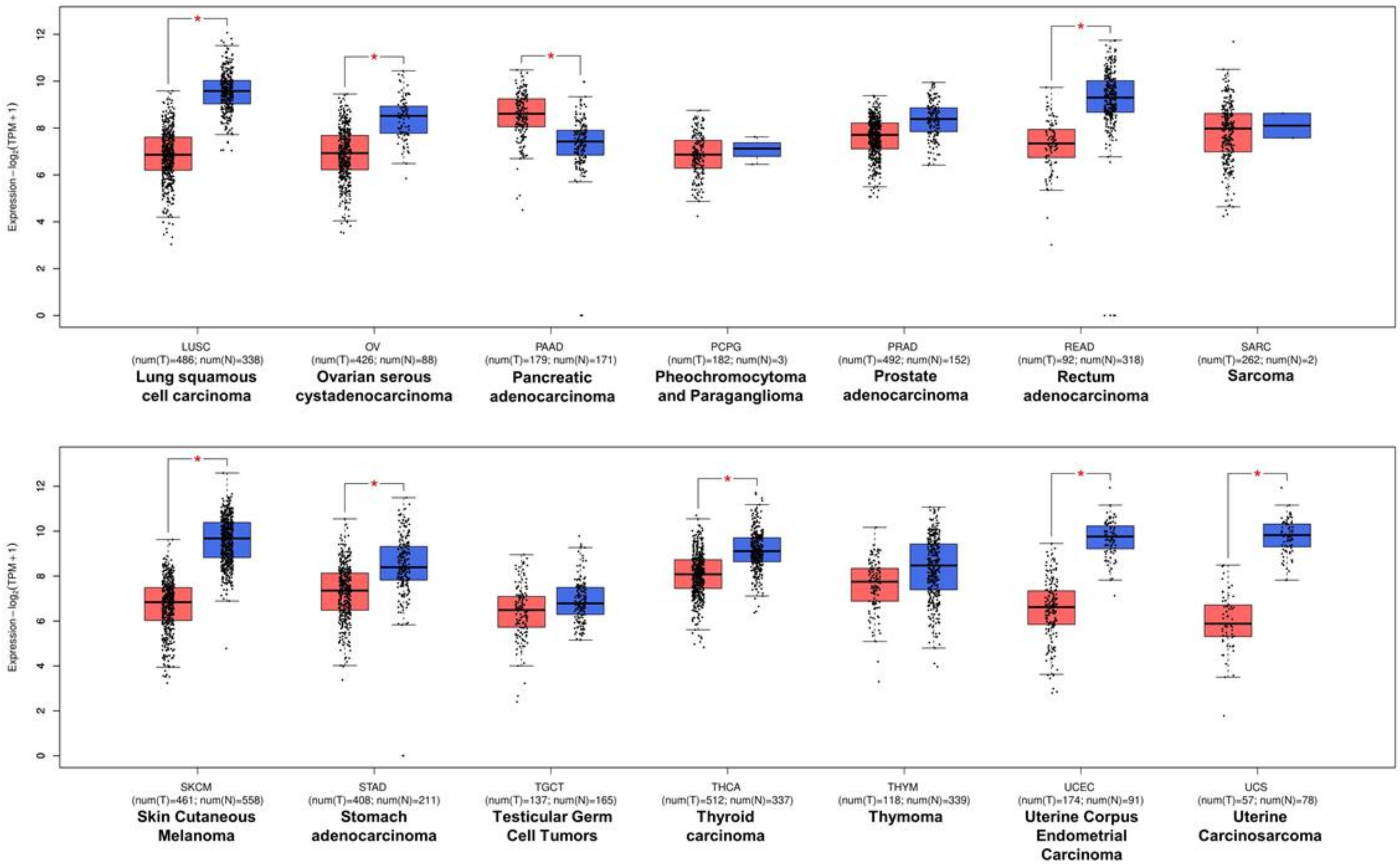


**Figure S2.** Boxplot diagrams of thioredoxin interacting protein (TXNIP) mRNA expression levels of tumor samples (red) and paired normal tissues (matched TCGA normal and GTEx data, blue). TPM = Transcripts Per Million. $|Log_2FC|$ Cut-Off = 1. * $p<0.01$.

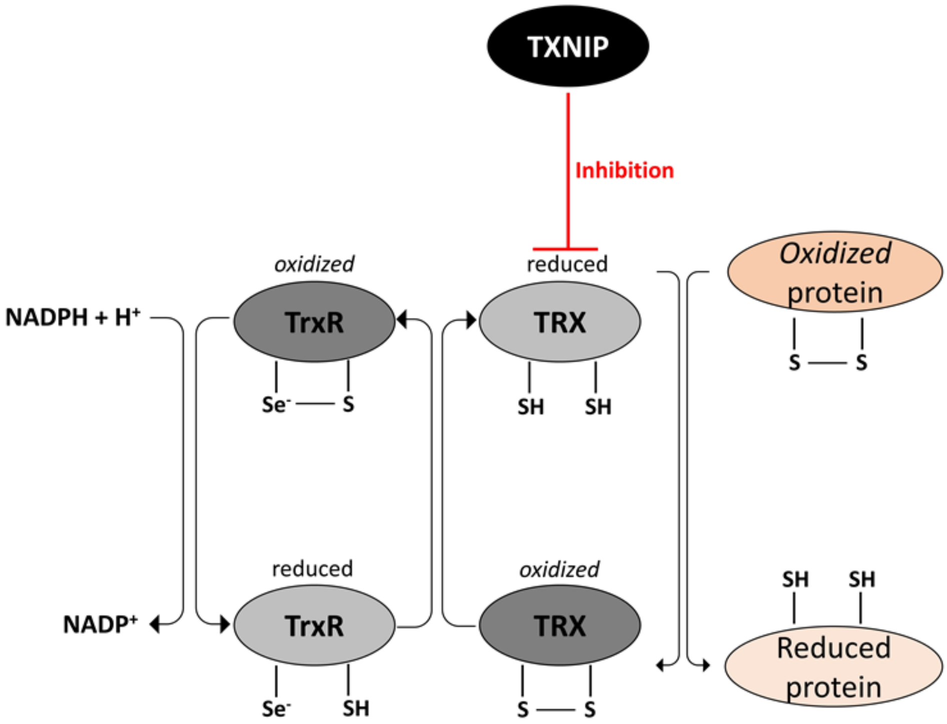


**Figure S3. The TRX/TXNIP system.** In its reduced form, oxidoreductase thioredoxin (TRX) catalyzes the reduction of oxidized cysteine residues and the cleavage of disulfide bonds of cellular proteins. TRX in turn is oxidized, and the reduced form is regenerated by TRX reductase (TrxR) via spending of NADPH. Thioredoxin interacting protein (TXNIP), the endogenous inhibitor of TRX activity, binds TRX only when it is in the reduced form.

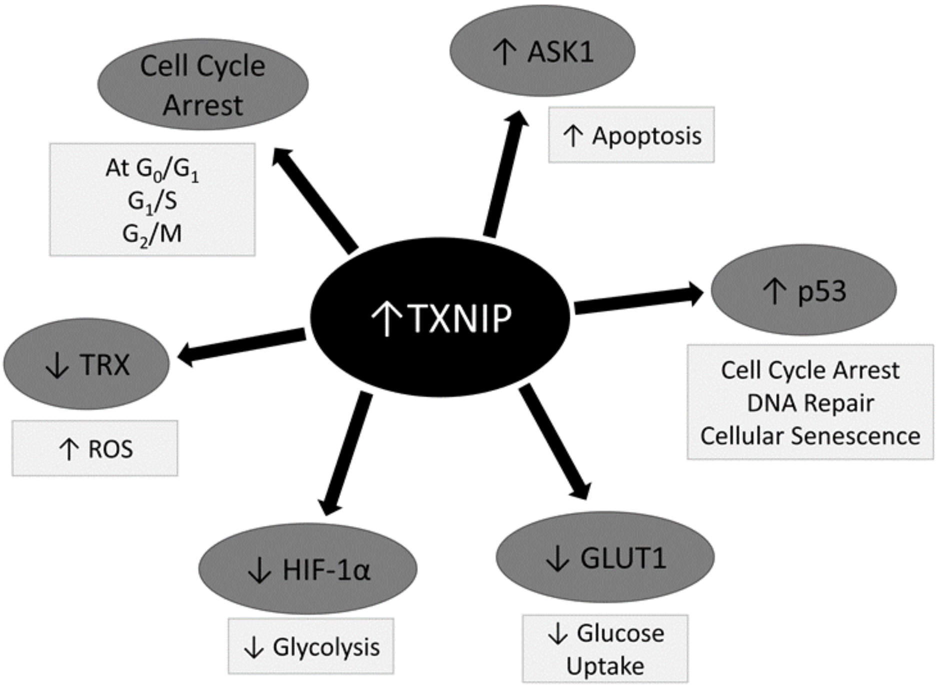


**Figure S4.** Thioredoxin interacting protein (TXNIP) is involved in tumorigenesis and cancer progression.

## References


1. Sheth, S.S.; Bodnar, J.S.; Ghazalpour, A.; Thipphavong, C.K.; Tsutsumi, S.; Tward, A.D.; Demant, P.; Kodama, T.; Aburatani, H.; Lusis, A.J. Hepatocellular carcinoma in Txnip-deficient mice. *Oncogene* **2006**, *25*, 3528–3536, doi:10.1038/sj.onc.1209394.
2. Nishizawa, K.; Nishiyama, H.; Matsui, Y.; Kobayashi, T.; Saito, R.; Kotani, H.; Masutani, H.; Oishi, S.; Toda, Y.; Fujii, N.; et al. Thioredoxin-interacting protein suppresses bladder carcinogenesis. *Carcinogenesis* **2011**, *32*, 1459–1466, doi:10.1093/carcin/bgr137.
3. Jeon, J.-H.; Lee, K.-N.; Hwang, C.Y.; Kwon, K.-S.; You, K.-H.; Choi, I. Tumor Suppressor VDUP1 Increases p27kip1 Stability by Inhibiting JAB1. *Cancer Res.* **2005**, *65*, 4485–4489, doi:10.1158/0008-5472.CAN-04-2271.
4. Morrison, J.A.; Pike, L.A.; Sams, S.B.; Sharma, V.; Zhou, Q.; Severson, J.J.; Tan, A.-C.; Wood, W.M.; Haugen, B.R. Thioredoxin interacting protein (TXNIP) is a novel tumor suppressor in thyroid cancer. *Mol. Cancer* **2014**, *13*, 62, doi:10.1186/1476-4598-13-62.
5. Kwon, H.-J.; Won, Y.-S.; Nam, K.-T.; Yoon, Y.-D.; Jee, H.; Yoon, W.-K.; Nam, K.-H.; Kang, J.-S.; Han, S.-U.; Choi, I.-P.; et al. Vitamin D3 upregulated protein 1 deficiency promotes N-methyl-N-nitrosourea and Helicobacter pylori-induced gastric carcinogenesis in mice. *Gut* **2012**, *61*, 53–63, doi:10.1136/gutjnl-2011-300361.
6. Feingold, P.L.; Surman, D.R.; Brown, K.; Xu, Y.; McDuffie, L.A.; Shukla, V.; Reardon, E.S.; Crooks, D.R.; Trepel, J.B.; Lee, S.; et al. Induction of Thioredoxin-Interacting Protein by a Histone Deacetylase Inhibitor, Entinostat, Is Associated with DNA Damage and Apoptosis in Esophageal Adenocarcinoma. *Mol. Cancer Ther.* **2018**, *17*, 2013–2023, doi:10.1158/1535-7163.MCT-17-1240.
7. Jung, H.; Kim, M.J.; Kim, D.O.; Kim, W.S.; Yoon, S.-J.; Park, Y.-J.; Yoon, S.R.; Kim, T.-D.; Suh, H.-W.; Yun, S.; et al. TXNIP Maintains the Hematopoietic Cell Pool by Switching the Function of p53 under Oxidative Stress. *Cell Metab.* **2013**, *18*, 75–85, doi:10.1016/j.cmet.2013.06.002.
8. Park, J.W.; Lee, S.H.; Woo, G.-H.; Kwon, H.-J.; Kim, D.-Y. Downregulation of TXNIP leads to high proliferative activity and estrogen-dependent cell growth in breast cancer. *Biochem. Biophys. Res. Commun.* **2018**, *498*, 566–572, doi:10.1016/j.bbrc.2018.03.020.
9. Kwon, H.-J.; Won, Y.-S.; Suh, H.-W.; Jeon, J.-H.; Shao, Y.; Yoon, S.-R.; Chung, J.-W.; Kim, T.-D.; Kim, H.-M.; Nam, K.-H.; et al. Vitamin D3 Upregulated Protein 1 Suppresses TNF-$\alpha$–Induced NF-κB Activation in Hepatocarcinogenesis. *J. Immunol.* **2010**, *185*, 3980–3989, doi:10.4049/jimmunol.1000990.
10. Dutta, K.K.; Nishinaka, Y.; Masutani, H.; Akatsuka, S.; Aung, T.T.; Shirase, T.; Lee, W.-H.; Yamada, Y.; Hiai, H.; Yodoi, J.; et al. Two distinct mechanisms for loss of thioredoxin-binding protein-2 in oxidative stress-induced renal carcinogenesis. *Lab. Invest.* **2005**, *85*, 798–807, doi:10.1038/labinvest.3700280.
11. Zhou, J.; Bi, C.; Cheong, L.-L.; Mahara, S.; Liu, S.-C.; Tay, K.-G.; Koh, T.-L.; Yu, Q.; Chng, W.-J. The histone methyltransferase inhibitor, DZNep, up-regulates TXNIP, increases ROS production, and targets leukemia cells in AML. *Blood* **2011**, *118*, 2830–2839, doi:10.1182/blood-2010-07-294827.
12. Tang, Z.; Kang, B.; Li, C.; Chen, T.; Zhang, Z. GEPIA2: an enhanced web server for large-scale expression profiling and interactive analysis. *Nucleic Acids Res.* **2019**, *47*, W556–W560, doi:10.1093/nar/gkz430.



© 2020 by the authors. Licensee MDPI, Basel, Switzerland. This article is an open access article distributed under the terms and conditions of the Creative Commons Attribution (CC BY) license (http://creativecommons.org/licenses/by/4.0/).
